# Supplementary figures and images for: Searching for biological feedstock material: 3D printing of wood particles from house borer and drywood termite frass
Source: PLoS One. 2021 Feb 19;16(2):e0246511. doi: 10.1371/journal.pone.0246511 (PMC7894899; doi:10.1371/journal.pone.0246511)

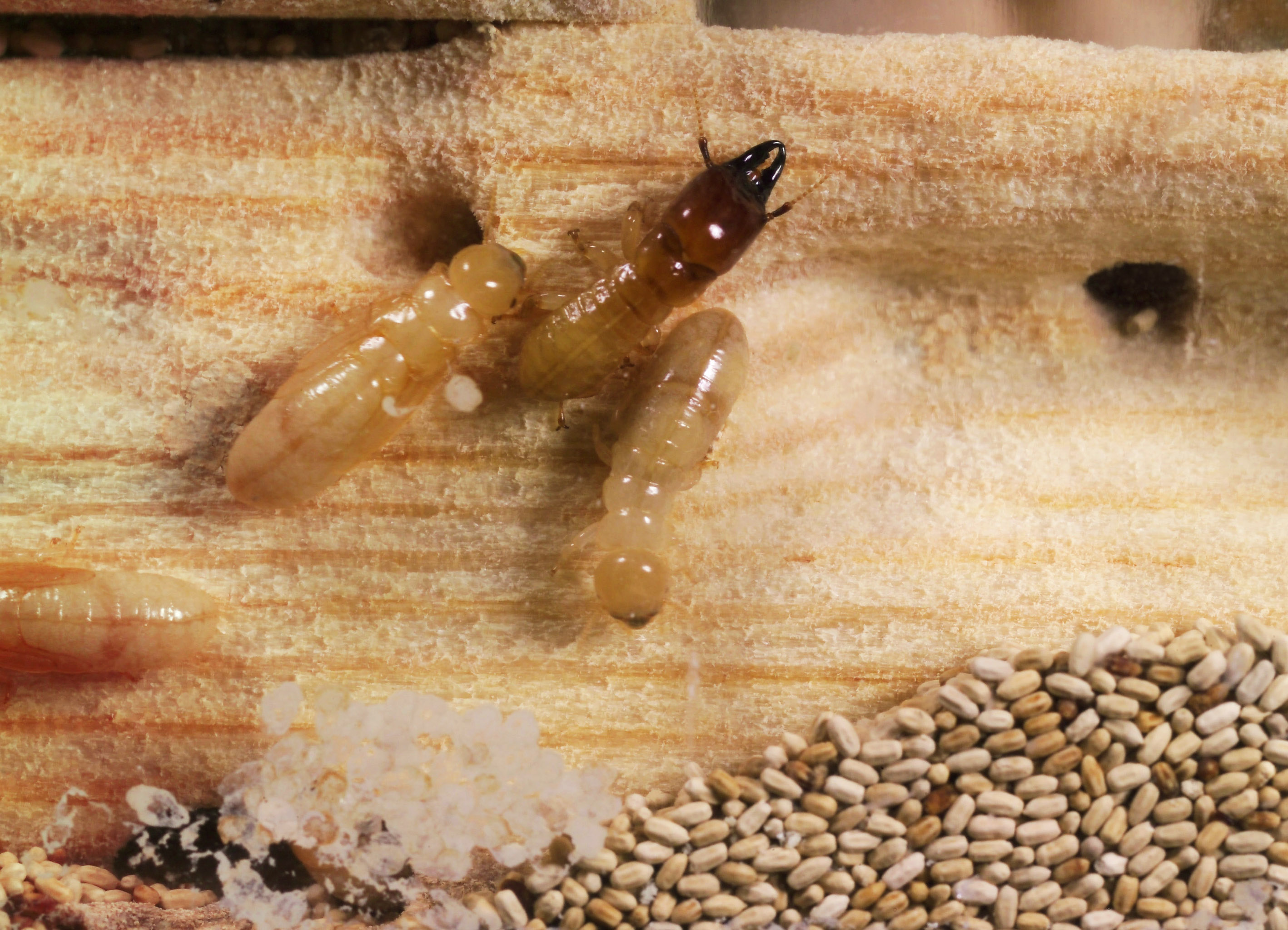

Supplement: S1 Fig — (JPG) [file pone.0246511.s001.jpg]

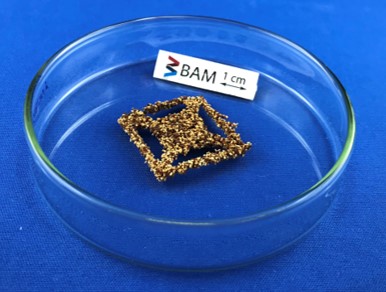

Supplement: S2 Fig — (JPG) [file pone.0246511.s002.jpg]

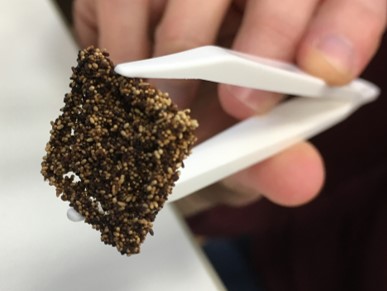

Supplement: S3 Fig — (JPG) [file pone.0246511.s003.jpg]

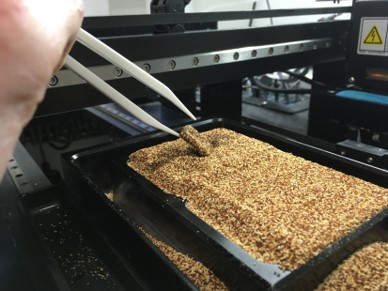

Supplement: S4 Fig — (JPG) [file pone.0246511.s004.jpg]

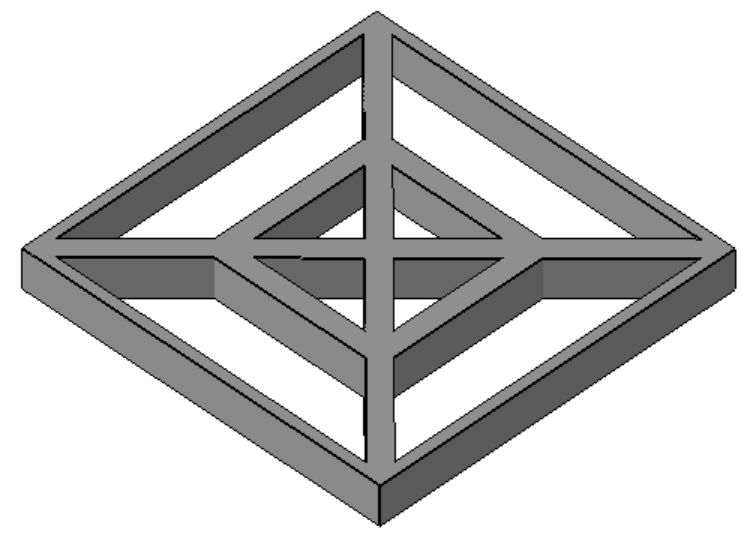

Supplement: S5 Fig — (PNG) [file pone.0246511.s005.png]

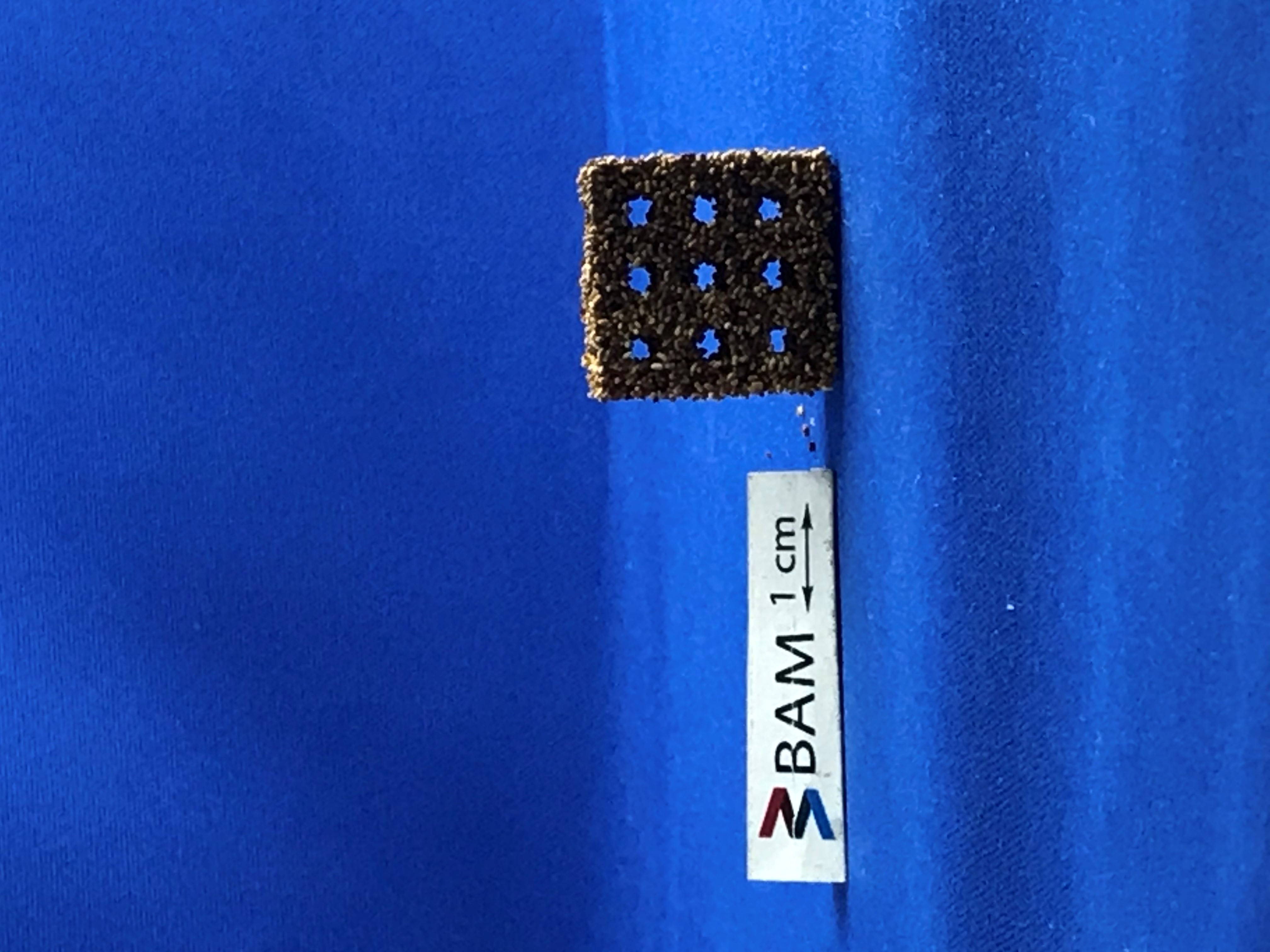

Supplement: S6 Fig — (JPG) [file pone.0246511.s006.jpg]

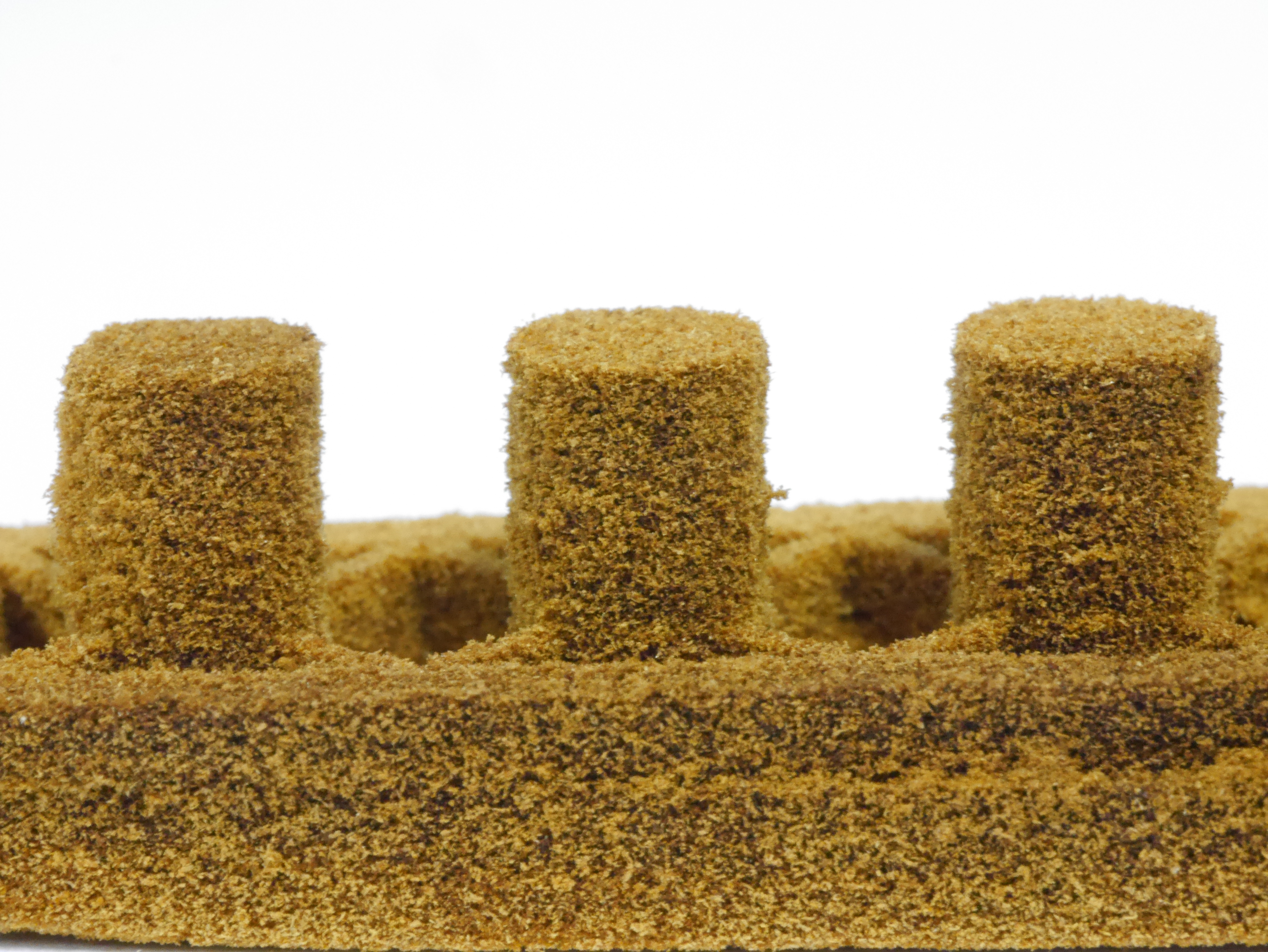

Supplement: S8 Fig — (JPG) [file pone.0246511.s008.JPG]

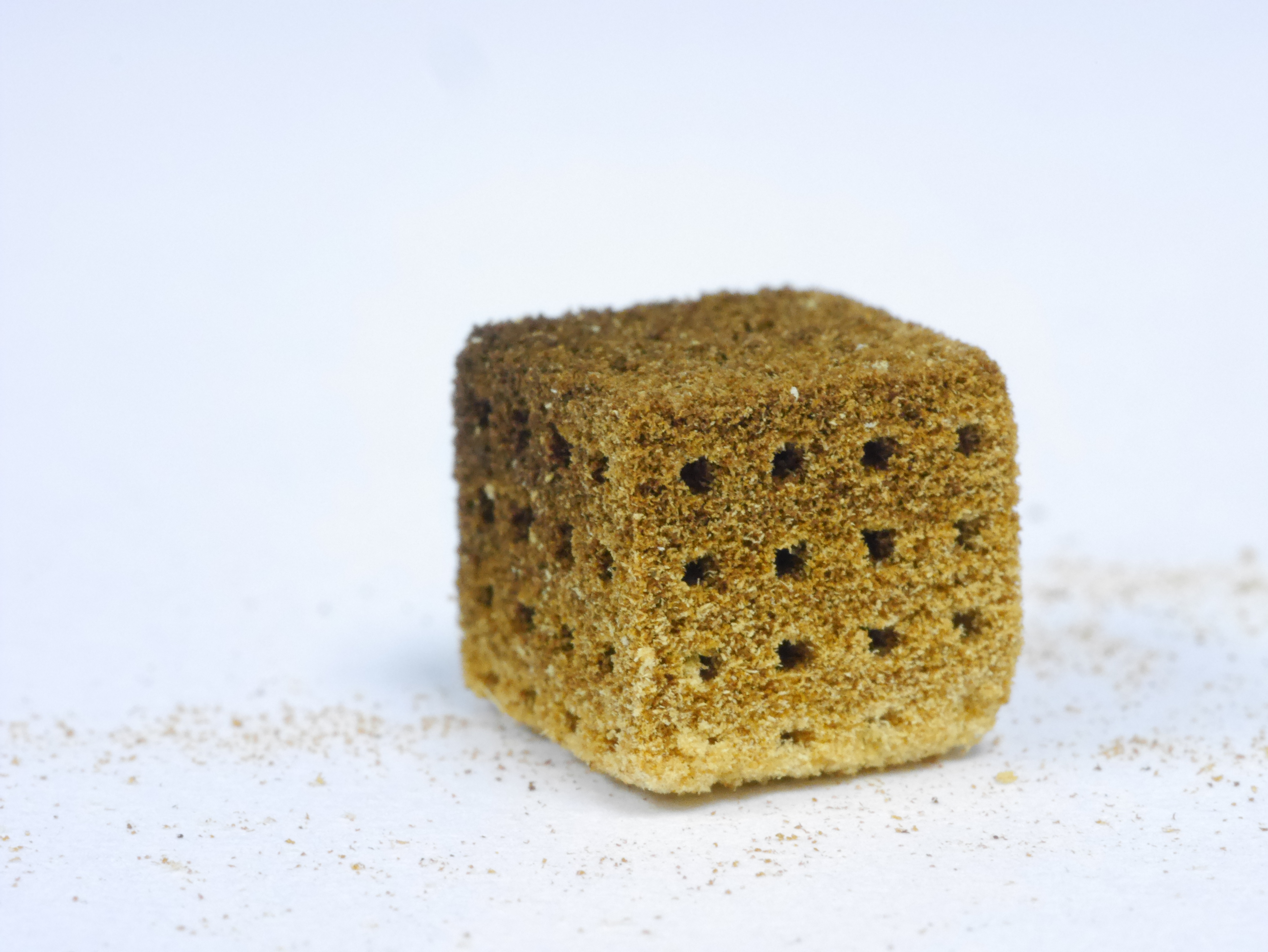

Supplement: S9 Fig — (JPG) [file pone.0246511.s009.JPG]
