# Supplementary material for: Searching for biological feedstock material: 3D printing of wood particles from house borer and drywood termite frass
Source: PLoS One. 2021 Feb 19;16(2):e0246511. doi: 10.1371/journal.pone.0246511 (PMC7894899; doi:10.1371/journal.pone.0246511)
Supplement: Attachment — Submitted filename: responsetoreviewers.docx [file pone.0246511.s013.docx]

“Searching for biological feedstock material: 3D printing of wood particles from house borer and drywood termite frass” by R. Plarre et al., which has been submitted as PONE-D-20-15678 already.

**Point by point response:**

1. Is the manuscript technically sound, and do the data support the conclusions?

The manuscript must describe a technically sound piece of scientific research with data that supports the conclusions. Experiments must have been conducted rigorously, with appropriate controls, replication, and sample sizes. The conclusions must be drawn appropriately based on the data presented.

Reviewer #1: Partly

Our reply: We have improved the introduction in that sense, that we now provided a detailed size analysis of about 200 termite frass particle. µCT measurements have been made on an ensemble of particles. It has been found that the ratio of length and width is 1.8 which is optimum for dense packing of particles.

We have added a figure 4: Fig 4. Rendered X-ray tomographic dataset of the termite pellets studied, 180 pellets (left). The length, breadth and width of each particle as determined from the 3D data (right). The average length of the pellets is around 1050 µm, its breath (or width) around 580 µm, yielding an aspect ratio of 1.8.

2. Has the statistical analysis been performed appropriately and rigorously?

Reviewer #1: No

Our reply: We have added a comprehensive statistical analysis of the termite frass particles based on µCT measurements. The flaky house borer frass was characterized by density measurements, but µCT measurements appear to be not target leading.

3. Have the authors made all data underlying the findings in their manuscript fully available?

The [PLOS Data policy](http://www.plosone.org/static/policies.action#sharing) requires authors to make all data underlying the findings described in their manuscript fully available without restriction, with rare exception (please refer to the Data Availability Statement in the manuscript PDF file). The data should be provided as part of the manuscript or its supporting information, or deposited to a public repository. For example, in addition to summary statistics, the data points behind means, medians and variance measures should be available. If there are restrictions on publicly sharing data—e.g. participant privacy or use of data from a third party—those must be specified.

Reviewer #1: No

Our reply: We have added µCT data of the termite frass particles, see point before, and have highlighted a new better printing result, improved figure 8 (former figure 7). Also, density and particle flowability measurements are consequently underlying the qualitative discussion of the usability of powder feedstocks considered.

New figure 8 (former figure 7): Fig 8a). Binder jetting powder bed filled with drywood termite frass, b),c) part printed according to figure 7, d) printed part similar to the one shown in Fig. 6.

4. Is the manuscript presented in an intelligible fashion and written in standard English?

PLOS ONE does not copyedit accepted manuscripts, so the language in submitted articles must be clear, correct, and unambiguous. Any typographical or grammatical errors should be corrected at revision, so please note any specific errors here.

Reviewer #1: Yes

5. Review Comments to the Author

Please use the space provided to explain your answers to the questions above. You may also include additional comments for the author, including concerns about dual publication, research ethics, or publication ethics. (Please upload your review as an attachment if it exceeds 20,000 characters)

Reviewer #1: This is an interesting topic but the manuscript appear to focus more on the process for 3D writing of wood particles from house borer and dry wood termite grass rather than evaluating the 3D printed material. There was no analysis conducted on the grass (e.g. ultimate analysis, proximate analysis). It is not clear why 3D printing has to be carried out if the 3D printed material is going to be eventually pyrolyzed. There was no statistical analysis. There was no mention of replication of what was done.

Our reply: We have discarded results associated to further processing of printed parts by pyrolysis and Si infiltration, as it highlights only one of the many possibilities of post processing. The manuscript is now clearly focused on introducing house borer and termites frass as naturally preprocessed feedstocks for 3D printing.
