# Supplementary material for: Searching for biological feedstock material: 3D printing of wood particles from house borer and drywood termite frass
Source: PLoS One. 2021 Feb 19;16(2):e0246511. doi: 10.1371/journal.pone.0246511 (PMC7894899; doi:10.1371/journal.pone.0246511)
Supplement: Attachment — Submitted filename: Response to Reviewers.docx [file pone.0246511.s014.docx]

Dear Editors,

We would like to thank the reviewers for the constructive comments and have seriously worked over the manuscript. Actions undertaken in response to the points raised by the reviewers have been listed one be one in the following.

Sincerely yours

Jens Günster

**Point by point response:**

Point raised by the reviewer: The experimental methodology for 3D printing needs be further detailed in the methodology section. Typically, the CAD models, print process parameters used for BJ need to be presented in the methods section. Currently, many are written in the results section.

Action undertaken by the authors: We have moved all CAD drawings of parts printed and their introduction to the Materials and Methods section.

Point raised by the reviewer: Also, please include which instruments you used to measure tap and flow properties of the powder within the methods section.

Action undertaken by the authors: We have added the following passage: “The tapped density was determined according to ISO 787-11 with a STAV 2003 type equipment from J. Engelsmann AG, Germany”

Point raised by the reviewer: As it stands there is intermixing of results and methods section and it goes back and forth within the results section. I understand that authors have used an iterative process of using smaller particles and larger particles to print two different geometry but putting the CAD models of these within the methods section will impart a flow that allows for the readers to know what the results section might have.

Action undertaken by the authors: Results and methods have been separated more consequently. In particular, the introduction of CAD models and printing parameters, as well as the introduction of µCT data have been moved from the results to the methods section.

Point raised by the reviewer: Also if in the results section, if authors could write the dimensional tolerance that they got from the printed parts. For eg. Fig. 5 and Fig 6 compares CAD and printed geometry. If the authors can just write how close was the printed pat when comparing it to Fig 5 and similarly for Fig 7 and Fig 8c within the text itself. Any measurement results will enable readers to understand with this novel process how well can you print your parts.

Action undertaken by the authors: Estimates of the dimensional tolerances of printed parts have been added in the results section.
